# Reviewed article: Carvalho AF, Guaranha DDFK, Marmett B, Reis JM, Rosa BS, Souza CLE, Dalcin TC, Amantea SL, Machado FV. A multicenter study on the mental health of Brazilian adolescent mothers, 2024. Epidemiol Serv Saude. 2025:34;e20240226.

**DOI:** 10.1590/S2237-96222025v34e20240226.a

**Published:** 2025-09-08

**Authors:** Elisabeth Meloni Vieira

**Affiliations:** 1 University of Sao Paulo Faculty of Public Health, Ciclos de Vida, Saúde e Sociedade

**Table:** 

Rating	Excellent	Good	Average	Below average	Poor
Interest	✓				
Quality		✓			
Originality		✓			
Overall		✓			

**Table:** 

Questionnaire	Yes	No	Not applicable
Does the manuscript contain new and significant information to justify publication?	✓		
Does the Abstract (Summary) clearly and accurately describe the content of the article?	✓		
Is the problem significant and concisely stated?	✓		
Are the methods described comprehensively?	✓		
Are the interpretations and conclusions justified by the results?	✓		
Is adequate reference made to other work in the field?	✓		

## Manuscript Structure

Length of article is: Adequate

Number of tables is: Adequate

Number of figures is: Adequate

Please state any conflict(s) of interest that you have in relation to the review of this paper (state “none” if this is not applicable): None

## Comments

O artigo apresenta um estudo multicêntrico desenvolvido em várias capitais brasileiras abrangendo as cinco regiões. Trata o relevante tema de avaliação da saúde mental de mães adolescentes, trazendo para a discussão o contexto social e cultural do processo saúde doença, em especial a saúde mental deste grupo de mulheres/meninas.

O artigo releva um estudo epidemiológico bem desenhado e realizado, com objetivos claros e bem definidos, o método detalhadamente apresentado e análise adequada. O uso do Instrumento DAS-21 é bastante apropriado para estudos semelhantes, pois se encontrada adaptado e validado no Brasil.

Os resultados são discutidos à luz de outros estudos semelhantes. No entanto, para a sua publicação seria necessária uma boa revisão de português. Há vários pequenos erros que prejudicam o texto e posso indicar como exemplo na P.2 linha 4; p.4 linha 13 ou p.7 linha 16.

